# Artificial intelligence literacy in Somali education: balancing opportunities, risks, and policy directions

**DOI:** 10.3389/frai.2026.1925433

**Published:** 2026-09-11

**Authors:** Ismail Mahad Hersi, Osman Elmi Omar, Abdinor Abukar Ahmed, Kowsar Osman Barre

**Affiliations:** 1 Center for Graduate Studies, Jamhuriya University of Science and Technology, Mogadishu, Somalia; 2 Benaadir Research, Consultancy and Evaluation Centre, Mogadishu, Somalia; 3 Department for Research and Development, Salaam University, Mogadishu, Somalia; 4 Somali Ocean Research Institute, Mogadishu, Somalia; 5 Department of Computer Science, East Africa University, Galkayo, Somalia

**Keywords:** academic integrity, AI literacy, artificial intelligence, equity, generative AI, policy, Somali education

## Abstract

Somalia’s education system continues to face severe constraints, including weak infrastructure, limited teacher support, and unequal access to learning resources. At the same time, generative artificial intelligence is increasingly entering educational practice through tools that can translate language, support writing, generate explanations, and extend tutoring beyond classroom hours. This Perspective argues that Somalia should respond neither by banning AI nor by adopting it without safeguards. Instead, AI literacy should be integrated as a foundational competency across secondary and tertiary education. Such an approach would help students use AI for language support, academic drafting, research planning, and personalized learning while also enabling them to identify hallucinations, bias, plagiarism risks, and privacy concerns. Drawing on a critical synthesis of recent peer-reviewed literature and policy documents, the article outlines the opportunities, risks, and implementation priorities for responsible AI adoption in Somalia. It shows that effective integration requires teacher professional development, curriculum revision, assessment reform, and clear ethical guidance. By combining opportunity with preparedness, Somalia can turn AI into a tool for educational inclusion and future readiness rather than a source of inequality or academic harm.

## 1 Introduction

Artificial intelligence is moving rapidly from a specialist technology to a general educational utility that shapes how learners search for information, draft texts, translate content, and receive feedback (UNESCO, 2021, 2023a). For Somalia, this shift matters because the education system continues to contend with conflict legacies, weak infrastructure, limited connectivity, and teacher shortages that constrain learning quality and access (UNESCO, 2023b, 2024, 2025b). In such a setting, AI may appear attractive because it can extend explanation and support beyond the traditional classroom. Yet the same tools can also intensify cheating, misinformation, privacy risks, and unfair access if they are adopted without policy direction (Alkaissi and McFarlane, 2023; Dwivedi et al., 2023; Cotton et al., 2024). The question, therefore, is not whether AI will enter Somali education. It already has. The real question is whether Somalia will treat AI as an unregulated convenience or as a literacy that must be taught, supervised, and governed (Laupichler et al., 2022; Alkaissi and McFarlane, 2023).

This Perspective takes the second position. It argues that AI literacy should become a foundational cross-disciplinary competency in Somali secondary schools and universities, because students need more than access to AI tools; they need the critical judgment to use those tools well (Laupichler et al., 2023; European Commission, 2025). Somalia’s education system remains shaped by long-standing challenges of access, quality, governance, and teacher capacity, and UNESCO’s Somalia brief continues to frame the sector around those priorities (MoECHE, 2024; UNESCO, 2024). Workshop on Somalia’s ICT in Education Policy and Masterplan specifically identified leadership, infrastructure, digital literacy, and teacher skills as the central policy issues for digital learning reform (UNESCO, 2023a, 2023b). At the same time, ITU data indicate that only 27.9% of individuals in Somalia used the internet in 2024 and 47.9% owned a mobile cellular telephone, which means any AI policy must assume uneven access rather than digital abundance (DataHub, 2025).

Peer-reviewed literature now treats AI literacy as a measurable educational competency rather than a vague technological preference, and recent systematic reviews report growing agreement on its definition, teaching tools, and effective pedagogies (Lintner, 2024; Biagini, 2025; Zhang et al., 2025). Recent studies show that AI literacy should be taught through hands-on learning, ethical reflection, and assessment-oriented practice, especially because students and teachers need to question AI outputs rather than accept them passively (Dilek et al., 2025; Erdem Coşgun, 2025; Lin et al., 2025). Frontiers studies further show that structured teacher training improves AI literacy, confidence, and attitudes toward AI integration, which makes educator preparation a core part of the policy response rather than an optional add-on (Bilbao-Eraña and Arroyo-Sagasta, 2025; Gao and Wang, 2026; Lademann et al., 2026). In higher education, studies published by SAGE and Taylor & Francis indicate that generative AI is already in student and teacher practice, but effective and equitable use still depends on training, support, and institutional guidance (Laru et al., 2025; Lee et al., 2025; O’Dea et al., 2026).

This Perspective uses a critical synthesis of recent peer-reviewed research on AI literacy, generative AI in education, academic integrity, and teacher preparedness, together with official Somali education and digital-inclusion policy documents. Its purpose is not to report original survey data, but to argue that AI literacy should function as a practical cross-disciplinary policy response for Somali secondary and tertiary education. The research gap is that Somalia’s AI literature is still emerging, with a recent Frontiers study showing strong integration of AI into academic research practices while also highlighting the need for professional development and guidance (Abubakar and Adan, 2026). At the policy level, Somalia’s current education and digital strategies emphasize ICT literacy, teacher skills, and digital inclusion, but they do not yet provide a dedicated AI-literacy framework for schools and universities.

### 1.1 Opportunities of AI in Somali education

A first opportunity is language support and translation. A major educational challenge in Somalia is the language gap between the language of instruction and the linguistic repertoires of many learners. Generative AI can help bridge that gap by translating content, simplifying academic explanations, and offering multilingual clarification that makes texts more approachable (Kohnke et al., 2023). This matters in a context where access to high-quality bilingual materials is limited and where many learners depend on one teacher, one textbook, or no textbook at all (UNESCO, 2023b, 2024, 2025b). AI does not replace the need for Somali-language curriculum development, but it can help learners move more confidently between Somali and English while building academic vocabulary and comprehension (Kohnke et al., 2023; UNESCO, 2023a).

In practical terms, language support matters because it affects every subject. A learner who cannot decode a textbook in chemistry, economics, or history will struggle regardless of motivation. AI can generate bilingual explanations, glossary lists, and simpler rephrasings that reduce cognitive load while teachers continue to teach core concepts. In this way, AI works best as a bridge tool that expands access to content already taught in class, rather than as a substitute curriculum (Xiang, 2024).

Beyond translation, AI can support academic writing and research assistance. Generative AI can function as a writing companion that helps students outline arguments, refine sentences, check grammar, and organize ideas before submission (Chan and Hu, 2023; Kasneci et al., 2023; Chiu, 2024). For students who are learning to write academic English or navigate scholarly conventions for the first time, that support can reduce anxiety and improve participation in academic life (Chan and Hu, 2023; Kasneci et al., 2023; Tlili et al., 2023). AI can also help with topic scoping, preliminary literature exploration, and summarization of complex material, provided students are trained to verify what the model produces rather than treat it as a final authority (Dwivedi et al., 2023; Gao et al., 2023). In low-resource environments, this kind of mediated support is especially valuable because it lowers the entry barrier to research and academic communication (Chen et al., 2020).

Another important opportunity is personalized feedback and tutoring. One of the most important promises of AI in education is the ability to provide immediate, individualized feedback at scale (Ouyang and Jiao, 2021; Tlili et al., 2023). Adaptive systems and large language models can explain a concept repeatedly, present alternative examples, and respond to a learner’s misunderstanding outside the formal lesson schedule (Kung et al., 2023). This is especially relevant in Somalia, where many classrooms face high student-to-teacher ratios and where one-to-one tutoring is often unrealistic (UNESCO, 2023b, 2024, 2025b). Used carefully, AI can serve as a supplemental study partner that extends learning time and helps students practice at their own pace without displacing the teacher (Adams et al., 2023; Kasneci et al., 2023; Laupichler et al., 2023).

The value of this supplemental role is that it can bring consistency to support that is otherwise uneven. A teacher may not always be available after class, and many students cannot afford private tutoring. An AI companion can answer repeated questions, provide extra examples, and offer low-stakes practice. However, this benefit is strongest when teachers design authentic, assessment-linked tasks that require students to compare AI outputs with course materials, because current reviews show that AI-supported learning works best through teacher mediation, prompt feedback, and structured tasks rather than passive use (Alfarwan, 2025; Alghamdi and Alghizzi, 2025; Corbin et al., 2025; Xiaoyu et al., 2025).

At the institutional level, AI can also reduce some of the routine burden carried by teachers and administrators. It can assist with lesson planning, question generation, feedback drafting, and basic administrative tasks such as organizing class materials or summarizing student progress (Holmes et al., 2019). Such support matters because overloaded teachers often have little time for formative feedback or differentiated instruction (Zawacki-Richter et al., 2019). However, the gains are real only when teachers are trained to use the tools critically and when human judgment remains in charge of pedagogical decisions (Crompton and Burke, 2023).

Finally, AI-assisted localization can make global learning content more usable in Somali contexts by adapting explanations, examples, and vocabulary to local needs (UNESCO, 2023b, 2024, 2025b). Across other African contexts, AI combined with human editorial oversight has already been used to accelerate the creation of localized educational content, including materials in languages that historically lacked sufficient textbooks (UNESCO, 2023b, 2024). For Somalia, that means AI can support—not substitute for—efforts to expand open educational resources, digitize content, and improve the relevance of learning materials (UNESCO, 2021, 2023a).

### 1.2 Risks and challenges of AI adoption

The most immediate educational risk is not simply error but dependency. If students use AI to generate answers before they think through a problem, they may bypass the mental effort that produces durable understanding (Dwivedi et al., 2023; Kasneci et al., 2023). Large language models are designed to produce plausible responses, which can create the illusion of mastery even when the underlying reasoning is shallow or incomplete (Alkaissi and McFarlane, 2023; Gao et al., 2023). In Somalia, where foundational learning already needs strengthening, uncritical AI reliance could worsen surface learning rather than deepen it (Holmes et al., 2019). Dependency becomes more dangerous when students are rewarded only for final answers rather than for showing thinking, drafts, or sources. If schools want AI to support learning, assessments must make room for explanation, reflection, and oral defense. Otherwise, students will naturally optimize for the quickest path, and the quickest path will often be AI-generated completion rather than genuine understanding.

A second risk concerns academic integrity and plagiarism. Generative AI creates new forms of misconduct because students can submit text, ideas, or solutions that are not genuinely their own (Dwivedi et al., 2023; Cotton et al., 2024). This is not only a detection problem; it is a learning problem, because hidden AI use can distort assessment, weaken skill acquisition, and erode trust between students and institutions (Crompton and Burke, 2023). The policy response must therefore include disclosure norms, assessment redesign, and explicit rules on what counts as permissible assistance (Cotton et al., 2024).

A third challenge involves hallucinations, misinformation, and bias. Large language models can generate fluent but false content, a phenomenon widely described as hallucination (Gao et al., 2023; Kung et al., 2023). They can also reflect bias from their training data, especially when they are used in multilingual or culturally specific settings where the model has limited high-quality exposure (Adams et al., 2023; Kohnke et al., 2023). For Somali education, the implication is straightforward: AI should be treated as a draft-generation tool, not an epistemic authority (Alkaissi and McFarlane, 2023). Students must learn to cross-check AI output against books, teachers, and reliable sources before relying on it in coursework (Laupichler et al., 2022; Gao et al., 2023).

A further concern is digital inequality and privacy. AI can widen existing inequalities when only students with devices, internet access, electricity, or paid subscriptions can use it effectively (UNESCO, 2023b, 2024, 2025b). That possibility is especially serious in a country where rural access, household poverty, and infrastructure gaps remain significant educational barriers (UNESCO, 2025b). AI systems also raise privacy issues because they may collect student prompts, writing samples, and usage traces that should be protected through transparent governance (Adams et al., 2023). If Somalia adopts AI without safeguards, the country risks creating a new digital divide inside an already unequal system (Holmes et al., 2019).

Finally, institutional readiness and teacher capacity are decisive. Even a well-designed policy will fail if teachers, school leaders, and university staff are not prepared to interpret or supervise AI use (Holmes et al., 2019; Crompton and Burke, 2023). Educational institutions need guidance on acceptable use, staff development, infrastructure planning, and equity safeguards before AI becomes widespread in classrooms (UNESCO, 2021, 2023a). Without that preparation, AI will appear in practice anyway, but in a fragmented and ungoverned form (Holmes et al., 2019; Dwivedi et al., 2023).

In other words, the danger is not only misuse after policy failure; it is also the silent normalization of AI use without any educational framework. Students may already be prompting systems for homework help, translation, and essay drafting. If institutions ignore this reality, they lose the chance to shape norms around disclosure, data protection, source checking, and fair assessment. The longer policy waits, the more difficult it becomes to build responsible habits from the start.

**Figure 1:**
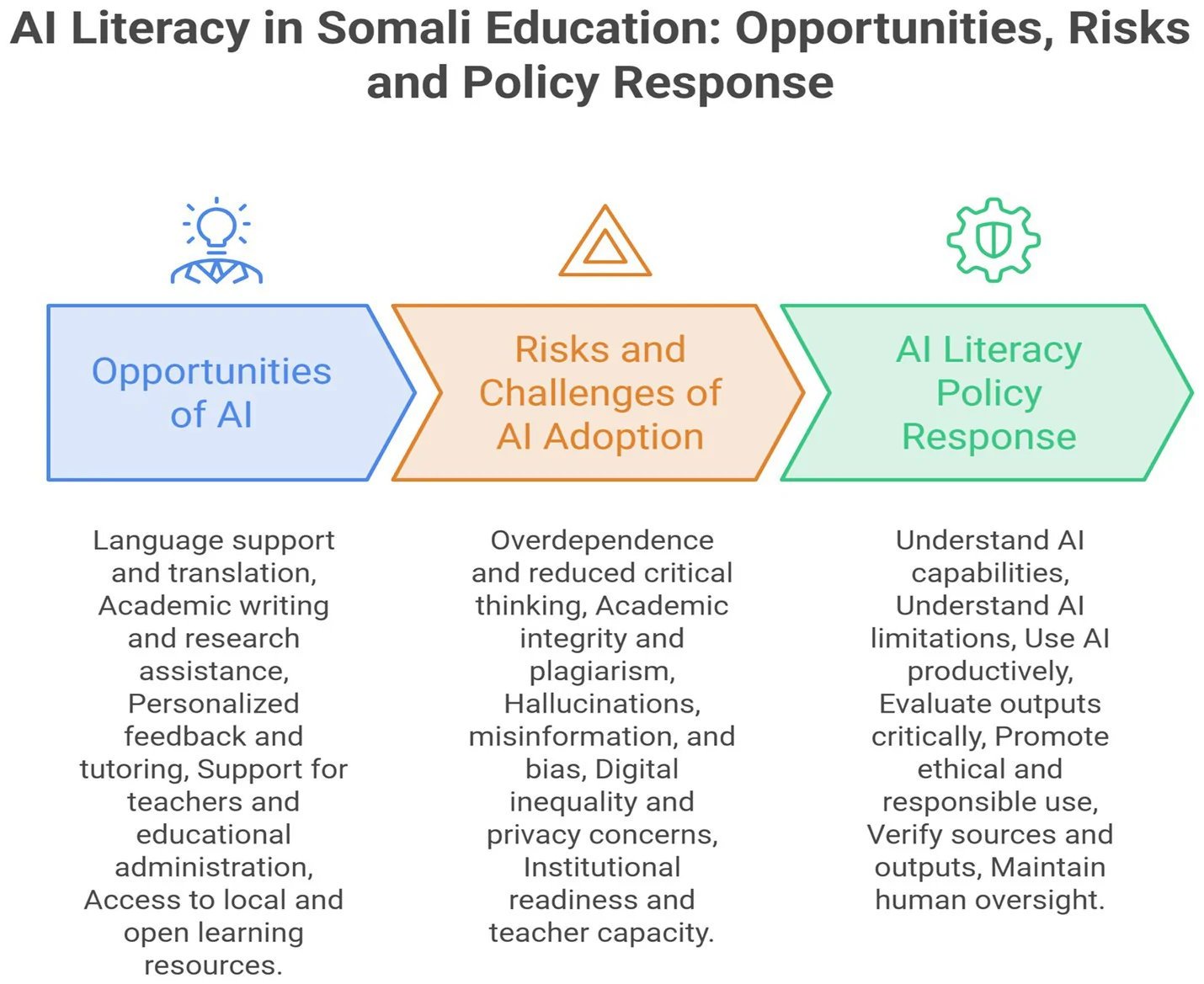
AI Literacy in Somali education: Opportunities, risks, and policy response.

Figure 1 summarizes the central argument of this Perspective by illustrating the relationship between the educational opportunities associated with artificial intelligence, the risks arising from its unregulated or inappropriate adoption, and the role of AI literacy as a policy response. While AI has the potential to enhance language support, academic writing, personalized learning, and access to educational resources, its adoption may also introduce concerns related to overdependence, academic integrity, misinformation, bias, digital inequality, and privacy. The framework therefore conceptualizes AI literacy as an integrating mechanism through which students and educators can develop the capacity to use AI effectively, evaluate its outputs critically, and apply it responsibly within educational settings. Consequently, the figure supports the article’s perspective that AI literacy should be considered a foundational component of Somalia’s approach to the safe, equitable, and productive integration of AI in education.

### 1.3 AI literacy as a policy solution

The proper response to these mixed outcomes is neither prohibition nor unmanaged enthusiasm. It is AI literacy (Long and Magerko, 2020). AI literacy can be defined as the capacity to understand what AI systems do, how they work at a basic level, what their limits are, how to use them productively, and how to evaluate their outputs ethically and critically (Laupichler et al., 2022; Laupichler et al., 2023). This is more than a technical skill. It is a civic and academic competency that prepares learners to make informed decisions about when to use AI, when to question it, and when not to rely on it at all (Laupichler et al., 2022; European Commission, 2025). For Somalia, that means AI should be taught as a cross-disciplinary literacy rather than a topic reserved for computer science departments (Zawacki-Richter et al., 2019; Ouyang and Jiao, 2021; Laupichler et al., 2023).

At secondary-school level, AI literacy can begin with age-appropriate modules on everyday AI, machine translation, prompt formulation, source verification, and responsible use in assignments (Long and Magerko, 2020; Kohnke et al., 2023). At university level, AI content should be embedded across faculties. Education students need to learn how AI changes assessment and tutoring; medical students need to understand safety, validation, and decision support; business and social science students need to examine data use, bias, and governance; and humanities students need to explore how AI reshapes language, authorship, and interpretation, as recent systematic reviews show that generative AI is already changing language-classroom practice and second-language acquisition (Bao et al., 2025; Lee et al., 2025; Liu et al., 2025; Lyu et al., 2025).

However, the design of AI-literacy policy for Somalia should be informed by emerging evidence from African education systems rather than relying predominantly on experiences from high-income countries. Evidence from Ghana, for example, shows that higher-education students recognize the academic benefits of generative AI while simultaneously reporting concerns about academic-policy violations, over-reliance, originality, security, and the absence of adequate training on safe and effective use (Baidoo-Anu et al., 2024). Evidence from South Africa further demonstrates both the opportunities and constraints surrounding generative AI in higher education, including potential benefits for teaching, learning, and research alongside challenges related to accessibility, connectivity, electricity, device availability, technical skills, and regulatory guidance (Modiba et al., 2025). These experiences suggest that AI literacy in Somalia should not be conceptualized simply as teaching students how to use generative AI tools; it should encompass critical evaluation of AI outputs, responsible use, awareness of limitations and bias, data protection, and the ability of educators and students to determine when AI assistance is educationally appropriate.

African-language representation provides an additional reason for contextualizing AI literacy. Research on low-resource East African languages shows that African languages remain substantially underrepresented in the natural-language-processing ecosystem and that developing usable language technologies requires the creation of high-quality text and speech resources (Nakatumba-Nabende et al., 2024). Research evaluating large language models across African languages has found substantial performance gaps relative to high-resource languages, including across text-generation and other language tasks (Ojo et al., 2025). More recent work demonstrates that targeted instruction tuning and African-language resources can improve model performance (Uemura et al., 2024). For Somalia, this has direct implications: AI literacy should include an awareness that the reliability of an AI system may vary according to language and context, and that students should not assume that an AI-generated response in Somali has the same evidentiary reliability as a response generated in a high-resource language. This reinforces the need for educational AI systems that increasingly incorporate African languages, locally relevant datasets, and culturally appropriate evaluation.

During the initial stages of implementation, foundational AI literacy modules could be delivered bilingually, using Somali to explain core AI concepts while progressively introducing English technical terminology. Such an approach could reduce language-related barriers to participation and support conceptual understanding in a multilingual, low-resource educational setting (Nakamura et al., 2023; UNESCO, 2025a). As students’ confidence develops, bilingual instruction can gradually transition toward greater use of English technical terminology while maintaining Somali as the primary medium for conceptual clarification during the early stages of implementation. Such staged language progression is consistent with current evidence on multilingual education and emerging frameworks for AI literacy implementation that emphasize scaffolded learning and gradual competency development (UNESCO, 2025a; Niri et al., 2026).

AI literacy, however, cannot substitute for institutional governance. The African Union’s Continental Artificial Intelligence Strategy promotes an Africa-centred, development-focused approach to AI that emphasizes ethical, responsible, and equitable development, national strategies, capacity building, and stronger regional and global cooperation, Importantly, the Strategy recognizes that AI systems should be adapted to Africa’s diverse languages, cultures, histories, and geographical contexts, while supporting the development of local capabilities in infrastructure, datasets, computing, research, and innovation (African Union, 2024). For Somalia, this implies that AI adoption in education should be accompanied by institutional governance mechanisms addressing privacy, transparency, accountability, human oversight, bias mitigation, and responsible procurement. AI literacy and AI governance should therefore be developed as complementary components: literacy equips students and educators to understand and critically evaluate AI, while governance establishes the institutional conditions under which AI can be used safely, fairly, and in ways that remain responsive to Somali educational and social realities. Complementing this regional policy direction, Effoduh et al. argue that trustworthy AI governance in Africa requires human-centred data governance, protection of privacy and personal data, fairness and accountability, representative and interoperable data, and transparent procurement when AI systems are obtained from external providers (Effoduh et al., 2024). These principles are particularly relevant to education, where AI systems may process student information, generate recommendations, influence assessment, or shape access to educational opportunities. Accordingly, Somalia’s AI-literacy agenda should be accompanied by institutional rules governing privacy, transparency, human oversight, responsible procurement, and mechanisms for identifying and addressing biased or inappropriate AI outputs. AI literacy and AI governance should therefore be developed as complementary components rather than as separate policy agendas.

Implementation should therefore combine parallel capacity development with differentiated levels of AI literacy rather than relying exclusively on a strictly sequential model in which secondary education is addressed first and universities only later. Secondary-school students and teachers could receive age-appropriate foundational modules covering AI concepts, responsible use, verification of AI-generated information, privacy, and academic integrity, while university students and faculty simultaneously receive more advanced training focused on research applications, disciplinary uses, critical evaluation, data governance, and institutional policy. This approach would allow capacity development to occur across educational levels while recognizing that the depth and application of AI literacy should differ according to learners’ roles and educational stages. Where infrastructure permits, these programmes could be supplemented by online and open-access learning resources. However, because connectivity, electricity, device access, and technical capacity remain important constraints in many African educational settings, digital delivery should complement rather than replace low-bandwidth, offline, and institution-based training approaches (Modiba et al., 2025). The objective should therefore be a coordinated national AI-literacy ecosystem in which capacity is developed across educational levels while remaining adapted to Somalia’s linguistic, infrastructural, and institutional realities.

In low-connectivity settings, AI literacy can initially be delivered through an offline-first implementation strategy. Foundational AI concepts may be introduced using printed instructional materials, teacher-led classroom demonstrations, downloadable educational resources, locally deployed language models where feasible, and structured prompt-writing activities before students transition to cloud-based AI systems (Tan et al., 2025; Aguilera et al., 2026). As connectivity improves, schools can progressively incorporate web-based generative AI tools, blended learning, and digital assessment while strengthening institutional capacity and reducing inequities through a phased implementation approach (Ali et al., 2026; Caspari-Sadeghi, 2026).

Teacher education is a prerequisite, not an afterthought. If teachers lack confidence with AI, they cannot guide students toward responsible use or distinguish productive assistance from prohibited substitution (Holmes et al., 2019; Chen et al., 2020; Adams et al., 2023). Teacher preparation should include practical demonstrations of how to use AI for lesson planning, how to detect suspiciously polished student writing, and how to design assignments that encourage comparison between AI output and human reasoning. Professional development should also create a shared vocabulary so that teachers, students, and administrators interpret responsible AI use consistently.

In practice, professional development may be delivered through short modular workshops, peer-learning communities, teacher mentoring, and mobile-based micro-learning activities that enable continuous capacity building while accommodating the realities of low-resource educational settings. These collaborative and sustained professional learning approaches enable teachers to progressively develop AI competencies, exchange classroom experiences, and translate AI literacy principles into everyday instructional practice (Kennedy and Beck, 2018; Liu et al., 2024; Amemasor et al., 2025).

Policy should therefore include teacher professional development, curriculum revision, student disclosure rules, and assessment reform that rewards critical thinking, oral explanation, project work, and source evaluation rather than only final text production (Cotton et al., 2024). Ethical guidance should also address privacy, fairness, accessibility, and cultural relevance so that AI use does not privilege already advantaged learners or erase Somali contexts (Adams et al., 2023).

This policy direction is consistent with UNESCO’s human-centered approach to AI in education, with OECD work on AI literacy, and with the European emphasis on ensuring that users of AI systems have adequate competence to understand opportunities and risks (UNESCO, 2021; European Commission, 2025). Such alignment matters because it shows that Somalia would not be inventing policy in isolation. Instead, it would be adapting globally recognized principles—human oversight, transparency, equity, and competence—to local conditions. That makes the policy more credible to universities, ministries, and development partners alike.

It is also realistic for a low-resource setting because curriculum integration and teacher development are more feasible than waiting for perfect infrastructure before taking action (UNESCO, 2023b, 2024, 2025b). A staged implementation would be more workable than a sudden nationwide overhaul. Somalia could begin with secondary-school awareness modules and teacher training pilots, then expand to university-wide foundational courses and faculty-specific applications. This gradual model would allow policymakers to test materials, refine guidance, and identify access gaps before full scale-up. It would also make it easier to adapt AI content into Somali and to align policy with local educational priorities.

## 2 Discussion

A productive debate about AI in education should move beyond the question of whether AI is good or bad. The more useful question is how education systems can use AI in ways that strengthen learning rather than weaken it (Dwivedi et al., 2023; Kasneci et al., 2023; Chiu, 2024).

Critics sometimes argue that AI literacy may distract from basic literacy and numeracy. That concern should be taken seriously, but it does not require an either-or choice. AI literacy can reinforce reading, writing, and analysis by making those skills more necessary, not less. The aim is to use AI as a scaffold for stronger fundamentals, while ensuring that the fundamentals remain central. That question is especially important in Somalia, where students already seek tools that can explain concepts, translate materials, and reduce the burden of limited support. If institutions refuse to address AI, students will use it informally and unevenly; if institutions embrace it without rules, they risk cheating, confusion, and inequity (Holmes et al., 2019; Cotton et al., 2024).

AI literacy provides the middle path. It acknowledges that students will encounter AI anyway, but it turns that encounter into a structured learning opportunity rather than a hidden practice (Long and Magerko, 2020; Laupichler et al., 2022; Laupichler et al., 2023). This has three advantages. First, it prepares learners for a future in which AI-mediated communication, assessment, and problem solving are increasingly normal in universities and workplaces (Ouyang and Jiao, 2021; European Commission, 2025). Second, it strengthens critical thinking by requiring students to assess AI outputs rather than accept them passively (Alkaissi and McFarlane, 2023; Kasneci et al., 2023). Third, it supports innovation because students who understand AI can adapt it to local problems instead of remaining only consumers of imported tools (Holmes et al., 2019; Crompton and Burke, 2023; Laupichler et al., 2023).

**Figure 2:**
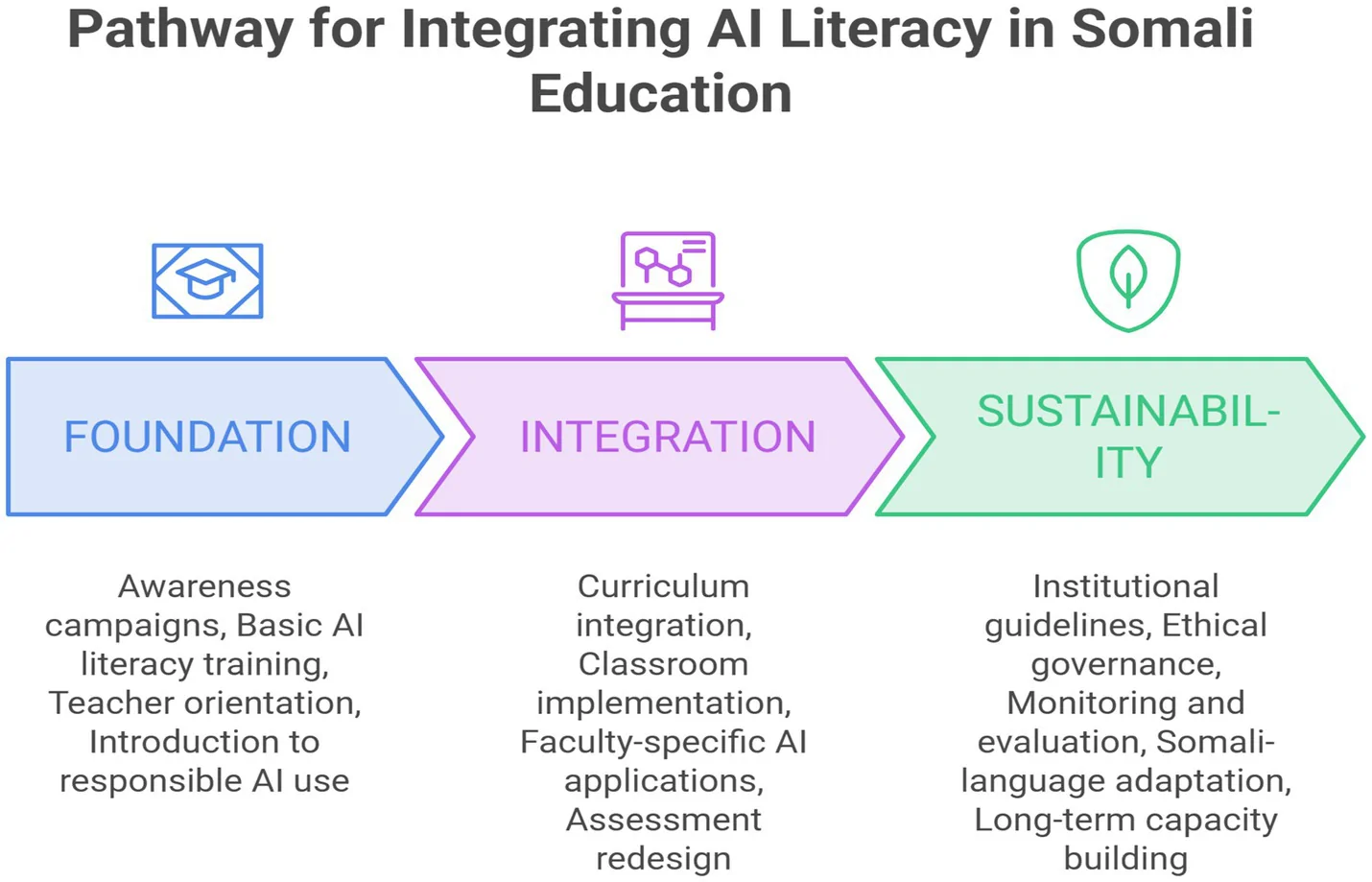
Pathway for integrating AI literacy in Somali education.

The policy is feasible because it does not require Somalia to solve every infrastructure problem before beginning. It requires curricular clarity, teacher development, ethics guidance, and gradual investment in access and connectivity (UNESCO, 2023b, 2024, 2025b). In that sense, AI literacy is not an optional extra. It is an educational safeguard and a development strategy (Figure 2).

Figure presents a phased pathway for integrating AI literacy into Somali education through three interconnected stages: foundation, integration, and sustainability. The framework suggests that successful AI adoption should begin with awareness campaigns, basic AI literacy training, teacher orientation, and responsible-use guidance before progressing to curriculum integration, classroom implementation, faculty-specific applications, and assessment reform. The final stage emphasizes long-term sustainability through institutional guidelines, ethical governance, continuous monitoring and evaluation, Somali-language adaptation, and capacity building. Collectively, the pathway highlights that AI literacy should be implemented as a gradual educational reform rather than a standalone technological intervention, ensuring that AI adoption remains aligned with educational quality, equity, and responsible governance objectives.

Taken together, the African evidence suggests that the feasibility of AI literacy in Somalia depends less on the immediate availability of advanced AI infrastructure than on the design of locally appropriate educational and institutional conditions. Experiences from Ghana and South Africa demonstrate that the educational value of generative AI can coexist with concerns about over-reliance, academic integrity, connectivity, electricity, device access, technical capacity, and regulatory guidance (Kamalov et al., 2023).

Evidence on African-language technologies further indicates that language representation and locally relevant data resources are important for reliable and equitable AI use (Nakatumba-Nabende et al., 2024; Ojo et al., 2025). Multilingual education guidance also emphasizes the importance of language-responsive approaches in educational systems, which is particularly relevant to Somalia given the role of language in access to learning (UNESCO, 2025a). At the policy level, the African Union’s Continental Artificial Intelligence Strategy and emerging African governance scholarship emphasize responsible, equitable, and context-sensitive AI development, including capacity building, data governance, transparency, and accountability (African Union, 2024; Effoduh et al., 2024). These findings strengthen the proposed Somali model by suggesting that AI literacy should be implemented alongside teacher capacity development, multilingual support, institutional governance, and low-bandwidth or offline delivery mechanisms rather than as a technology-only intervention (Aguilera et al., 2026). The proposed Foundation–Integration–Sustainability pathway therefore represents an adaptable policy direction rather than a fixed implementation sequence, allowing institutions to adjust the depth and pace of implementation according to infrastructure, institutional capacity, and learner needs.

In the Somali context, the sustainability phase should be aligned with successive implementation cycles of the National Education Sector Strategic Plan 2023–2027 and future education-sector reforms, allowing AI literacy policies to be periodically reviewed, updated, and strengthened as technological and educational needs evolve.

This Perspective has several limitations. First, it is conceptual and policy-oriented rather than empirical and does not present original data on AI use among Somali students, teachers, or educational institutions. Second, evidence regarding AI adoption in Somalia remains limited, requiring reliance on international literature and broader educational policy frameworks. Future studies should examine AI awareness, literacy levels, patterns of use, academic integrity concerns, and educator preparedness across Somali schools and universities to provide locally grounded evidence for policy development. Furthermore, because AI technologies continue to evolve rapidly, national AI literacy policies should remain adaptive and periodically updated to reflect technological, educational, and ethical developments.

## 3 Conclusion

Artificial intelligence has a positive future in Somali education, but only if it is guided by literacy, ethics, and policy rather than left to improvisation. The evidence reviewed in this Perspective shows that AI can support language learning, writing, tutoring, and teacher workload, while its risks include overdependence, plagiarism, hallucination, bias, and inequality. Somalia’s most defensible response is therefore to embed AI literacy across secondary schools and universities, strengthen teacher preparation, protect student data, and establish clear rules for responsible use. Such an approach will not solve every educational challenge, but it can help ensure that AI serves inclusion, quality, and preparedness rather than confusion and harm. In that sense, AI literacy is not merely a response to a new tool. It is a practical foundation for shaping a generation of learners who can engage the digital world with judgment, confidence, and responsibility.

## Data Availability

The original contributions presented in the study are included in the article/supplementary material, further inquiries can be directed to the corresponding author.
